# Healthful Houses

**Published:** 1872-02

**Authors:** 


### HEALTHFUL HOUSES.

"Ventilation, and other Requisites to a Healthy and Comfortable Dwelling," by Ross Winans, of Baltimore, is a tract of 43 pages, written by the great millionnaire of the Monumental City, for distribution among the people, for their instruction in the building and purchase of houses best calculated to preserve and promote the health of the family. Mr. Winans is building a hundred dwellings, each calculated to afford accommodations to one or two families of persons of moderate means, where coziness, comfort, and health can be secured, at a very moderate rent; thus are the rich men of our country, such as Peabody, and Stewart, and Winans, inaugurating a new system of public benevolences, in the expenditure of their millions for the highest benefit of those who are honorably struggling to secure and maintain a business footing in the world. These are men of elevated and far-reaching views; they do not propose to spend the money which they have made by their individual enterprise, integrity, and energy, in making a few rich, by free gifts, but in helping the many to live in comfort on a small portion of their earnings, thus enabling them to save something to increase their investment, or to extend their business, and in a way not to degrade, by accepting a free gift, but to elevate and make them feel their independence, by paying all that was asked, for what they get.

While Mr. Winans is thus helping clerks and others with small families to have homes, with elevated surroundings, at moderate rates, the prince of New York merchants is now completing a building whose erection we have pleasantly watched for two years, day by day, from our chamber window. It will be completed in a few months, at the expense of several millions of dollars; large enough to give shelter to fifteen hundred persons. There is no building more imposing in all New York city. It fronts on a whole block, and is eight stories high, built of iron and brick. The object of this gigantic structure is to afford to girls and women, who have to depend on their own individual exertions for a living, a tidy, comfortable place of abode. Most of the rooms are single, some double, for the accommodation of sisters, or mother and daughter; besides these rooms, there are parlors, dining-rooms, libraries, pianos, and other facilities for social amusement, improvement, and recreation.

The rooms are to be all lighted, warmed, and ventilated, after the latest improvements, and will be afforded at a fixed weekly rent, at a price proportioned to the size and elevation of the apartment.

The renters can take their meals elsewhere, or order them in the establishment; each pays for what is ordered and consumed, making the cost of a meal from five cents upwards. The provisions will be placed on the table, as near as possible at the actual cost; the aim being to bring the entire cost of board, fuel, room rent, gas, and washing, as little as possible,—under three dollars a week. Those who wish to live at a higher rate can do so, if they pay for it; everything must be paid for on the spot; there will be no encouragement to get into debt.

It is not a charitable institution. It is a regular hotel, only for a certain class of persons,—industrious, friendless women, who have no husbands, or brothers, or parents, to help them, but who are disposed to help themselves in a respectable way, by honest work.

As at any other hotel, they can leave when they please, return when they please, if they only pay for what they get, and keep good hours, good company, and work for the means to pay their way at the house. No person will be admitted at any price who has other means of support than the work of their own hands.

The lower portion of the building is for stores, some thirty or forty; these will be rented out, and the proceeds will go to paying the expenses of the establishment as far as possible, so as to reduce the rent to the lowest cent.

Very few persons outside of New York have any adequate idea of the magnificent and far-reaching character of this enterprise.

There are thirty thousand girls and women in New York who have to sew for a living. Some good dressmakers command two dollars and a half a day and their board; but the average is not over a dollar and a half a day, making nine dollars for six working-days, provided they are always well, and have as much work as they can do; but in a year's labor there is perhaps an average of seven dollars a week. Rooms on the fourth floors, or garrets in out-of-the-way streets, command three dollars a week, without fuel, gas, and washing; here, then, are women who must dress and feed themselves, and pay for washing, fuel, and lights, with four dollars a week. Having to purchase coal "by the small," it costs them about twenty dollars a ton. To get to their out-of-the-way lodgings they will, after six o'clock in the evening, which is quite dark in winter in New York, have to pass through long, narrow, dark, filthy, and muddy streets; the sidewalks and grocery corners crowded with boisterous, swearing, beer-drinking, cigar-smoking young men, with their low witticisms, and profane exclamations, and obscene remarks, when a decent-looking woman passes along. Through such a gauntlet do the respectable sewing-girls and women of New York city have to pass, by thousands, every night of every week, of every month in the year. Reader, what a terrible thought it would be, that your daughter, or mine, or their daughters, should ever have to pass such a fiery trial; and then, when they get to their cold rooms, dark, desolate, and dreary, with no smile of welcome to greet them, but to sit there hour by hour until bedtime comes, all alone; it is dreadful to think of. These are the very dangers and difficulties which A. T. Stewart is seeking to obviate — to give industrious, respectable young girls all the comforts of a tidy home, for three dollars a week, in a healthy and respectable locality, within one block of the finest street of its size in New York city, Fifth Avenue not excepted; that is, the extension of Fourth Avenue, called Park Avenue, from 34th Street to 42d Street, ending against the Grand Union Depot. With such a home, all they can earn over three dollars a week, they can lay away in a savings-bank, bearing interest, deducting only what they have to spend for dress and trivial incidental expenses.

You cannot estimate the elevating influence which such surroundings must have on young girls. The right to enter such a house, as their home, elevates; the sight of the fine apartments elevates; association with well-dressed and well-behaved persons elevates; and so does the piano, the harp, the library, the reading-room, for all these are free; besides, there will be regular series of lectures on the popular, useful topics of the day, and concerts, and reunions, all under the same roof, not requiring any attendant.

We verily believe that this is one of the most important enterprises of modern times, and it is commended in all its bearings to the thoughtful consideration of reflecting men, and is worthy of imitation in all crowded communities.

